# Leukemia development initiated by deletion of *RBP-J*: mouse strain, deletion efficiency and cell of origin

**DOI:** 10.1242/dmm.036731

**Published:** 2018-12-18

**Authors:** Brian Chipman Belyea, Fang Xu, Maria Luisa Soledad Sequeira-Lopez, Roberto Ariel Gomez

**Affiliations:** Department of Pediatrics, University of Virginia School of Medicine, MR4 Building, 409 Lane Road, Charlottesville, VA 22908, USA

**Keywords:** Leukemia, Notch signaling, Renin

## Abstract

Conditional deletion of *RBP-J*, the major transcriptional effector of Notch signaling, specifically within renin-expressing cells leads to the development of B-cell leukemia. However, the influence of contributing factors such as mouse strain, cell of origin and Cre recombinase copy number are unknown. In this study, we compared *RBP-J* deletion efficiency using one versus two copies of Cre recombinase. Further, we compared the incidence and timing of leukemia development in two unique strains of mice, C57BL/6 and 129/SV, as well as at different B-cell developmental stages. We found that animals expressing two copies of Cre recombinase developed B-cell leukemia at an earlier age and with more fulminant disease, compared with control animals and animals expressing one copy of Cre recombinase. In addition, we found a difference in leukemia incidence between C57BL/6 and 129/SV mouse strains. Whereas deletion of *RBP-J* in renin-expressing cells of C57BL/6 mice leads to the development of B-cell leukemia, 129/SV mice develop dermatitis with a reactive, myeloproliferative phenotype. The difference in phenotypes is explained, in part, by the differential expression of extra-renal renin; C57BL/6 mice have more renin-expressing cells within hematopoietic tissues. Finally, we found that deletion of *RBP-J* in Mb1- or CD19-expressing B lymphocytes does not result in leukemia development. Together, these studies establish that renin progenitors are vulnerable cells for neoplastic transformation and emphasize the importance of genetic background on the development of inflammatory and malignant conditions.

This article has an associated First Person interview with the first author of the paper.

## Abstract (precis)

**Summary:** Using a mouse model of precursor B-cell leukemia, we have investigated influencing factors on leukemia genesis, including genetic background, cell of origin and deletion efficiency.

## INTRODUCTION

The Notch signaling pathway is involved in a broad spectrum of cell-fate decisions, including self-renewal, lineage commitment and differentiation, within a variety of cell types. During hematopoiesis, the Notch pathway regulates lineage commitment decisions at various lymphocyte developmental stages (Tanigaki and Honjo, 2007). Multiple studies have demonstrated that Notch signaling promotes T-cell commitment over B-cell fates from bi-potent lymphocyte progenitors (Han et al., 2002; Pui et al., 1999; Radtke et al., 1999). Later, during B-cell development, Notch signaling regulates marginal zone versus follicular B-cell fates in the spleen (Tanigaki et al., 2002, 2003). Given these important roles during lymphocyte development, Notch signaling must be tightly controlled. Indeed, deregulated expression of Notch members can lead to the development of hematological malignancies, with activating mutations of *Notch1* occurring in more than half of cases of T-cell leukemia (Weng et al., 2004). In contrast, activation of the Notch pathway appears to cause growth arrest in a wide range of B-cell malignancies (Zweidler-McKay et al., 2005).

During skin development, the Notch signaling pathway plays multiple roles, including stem cell maintenance, progenitor-cell-fate specification, and differentiation within epithelial cells and hair follicles (Nowell and Radtke, 2013). Loss of Notch signaling in embryos leads to hair loss, epidermal hyperkeratinization and epidermal cyst formation (Yamamoto et al., 2003). Further, conditional deletion of Notch signaling within the skin during postnatal life results in aberrant proliferation and differentiation of epithelial cells within the epidermis, as well as degeneration of hair follicles into epidermal cysts (Dumortier et al., 2010). Finally, loss of Notch signaling in the epidermis results in chronic inflammation resembling atopic dermatitis (Dumortier et al., 2010; Demehri et al., 2008) and, in extreme cases, promotes tumorigenesis (Demehri et al., 2009).

Our laboratory previously demonstrated that conditional deletion of the Notch signaling effector *RBP-J* (also known as *Rbpj*) within a subset of B cells that express renin leads to the development of B-cell leukemia in mice (Belyea et al., 2014). However, whether deletion of *RBP-J* within other B-cell progenitors or in different strains of mice leads to leukemia development is unknown. In this work, we tested the hypothesis that the type of proliferative/neoplastic process resulting from *RBP-J* deletion is determined by deletion efficiency, genetic background and stage of differentiation of the cell of origin involved.

## RESULTS

### Influence of mouse strain and Cre recombinase copy number on leukemia development

Previously, we reported that conditional deletion of *RBP-J* within renin-expressing cells leads to a highly penetrant and aggressive form of precursor B-cell leukemia (Belyea et al., 2014). In these studies, our animals originated from a mixed background with both C57BL/6 (Bl6) and 129/SV (SV) strains used to generate control and mutant mice. To assess the influence of mouse strain on leukemia development, we generated control and mutant mice using two different renin-Cre animals: one generated in pure SV background mice, ‘Ren1^dCre^(SV)’, and another backcrossed for over 15 generations in Bl6 background mice, ‘Ren1^dCre^(Bl6)’. To study the effect of more efficient *RBP-J* deletion, we generated control and mutant animals with either one or two copies of Cre recombinase in both the SV and Bl6 backgrounds. We then monitored these animals for development of leukemia.

**Fig. 1.**
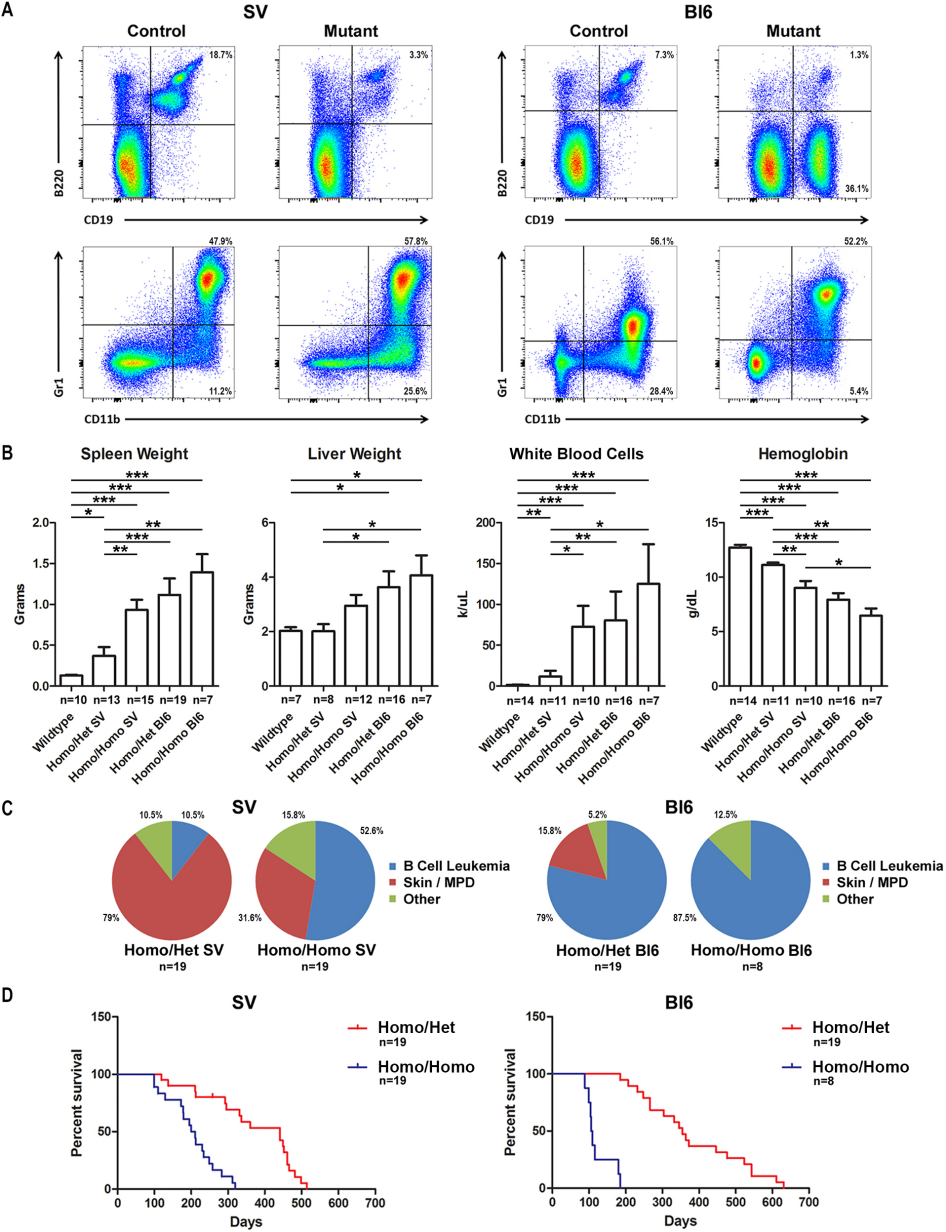
**Deletion of *RBP-J* within renin cells of Bl6 and SV mice leads to B-cell leukemia and MPD, respectively.** (A) Representative flow cytometry plots performed on the bone marrow of control and mutant mice from the SV (left panel) and Bl6 (right panel) background. Conditional deletion of *RBP-J* within renin cells of SV mice results in decreased number of CD19^+^B220^+^ B cells and an increase in CD11b^+^Gr1^−^ and CD11b^+^Gr1^+^ myeloid cells. Conversely, conditional deletion of *RBP-J* within renin cells of Bl6 mice results in an aberrant population of CD19^+^B220^dim^ leukemic B cells and a decrease in myeloid cells. (B) Mutant animals from the Bl6 background have increased spleen weight, liver weight and white blood cell count, as well as decreased hemoglobin, compared with mutant animals from the SV background. Further, mutant SV animals with two copies of Cre recombinase have increased spleen weight, increased white blood cell count and decreased hemoglobin compared with mutant SV animals with one copy of Cre recombinase. There was a trend towards worse disease in mutant Bl6 animals with two copies of Cre recombinase compared with Bl6 animals with one copy of Cre recombinase, although this was not statistically significant. *P<0.05, **P<0.01 and ***P<0.001, Mann–Whitney *U*-test. The sample size for each group is shown. (C) Animals with two copies of Cre recombinase (*RBP-J^fl/fl^;Ren1^dCre/Cre^*) have a higher incidence of B-cell leukemia compared with mice with only one copy of Cre recombinase (*RBP-J^fl/fl^;Ren1^dCre/+^*) for both the SV and Bl6 background. Mutant animals from the Bl6 background predominantly develop B-cell leukemia, whereas mutant animals from the SV background have a higher incidence of MPD (*P*<0.001, Fisher's exact test). The sample size for each group is shown. (D) In both SV and Bl6 mice, survival decreases in mutant animals with two copies of Cre recombinase (*RBP-J^fl/fl^;Ren1^dCre/Cre^*) compared with those with only one copy (*RBP-J^fl/fl^;Ren1^dCre/+^*). The sample size for each group is shown.

We found that animals with conditional deletion of *RBP-J* in renin cells from a Bl6 background primarily developed B-cell leukemia. Conversely, animals from an SV background primarily developed a severe myeloproliferative disorder (MPD). Immunophenotyping of bone marrow by flow cytometry demonstrated two distinct marrow phenotypes, including B-cell leukemia (B220^dim^CD19^+^), in the majority of Bl6 animals and a myelomonocytic (Gr1^+^CD11b^+^) phenotype in the majority of SV animals (Fig. 1A). Mutant animals from both strains showed marked splenomegaly, hepatomegaly, leukocytosis and anemia compared with controls; however, this was more severe in Bl6 mice. Bl6 mutants with one copy of Cre recombinase (‘Homo/Het Bl6’) had increased spleen weight [Mann–Whitney statistic (U)=35, B16 mutant (n_Bl6_)=19, SV mutant (n_SV_)=13, *P*<0.001], liver weight (U=27, n_Bl6_=16, n_SV_=8, *P*<0.05), white blood cell (WBC) count (U=35, n_Bl6_=16, n_SV_=11, *P*<0.01) and decreased hemoglobin (U=11, n_Bl6_=16, n_SV_=11, *P*<0.001) compared with SV mutants with one copy of Cre recombinase (‘Homo/Het SV’) (Fig. 1B). Further, Bl6 mutants with two copies of Cre recombinase (‘Homo/Homo Bl6’) had decreased hemoglobin (U=13, n_Bl6_=7, n_SV_=10, *P*<0.05) compared with SV mutants with two copies of Cre recombinase (‘Homo/Homo SV’).

In addition, animals with two copies of Cre recombinase developed B-cell leukemia at an increased frequency and also developed leukemia at an earlier age compared with animals with one copy of Cre recombinase (Fig. 1C,D). They also showed more severe disease, with a trend towards increased spleen weight, liver weight and WBC count, and decreased hemoglobin, compared with animals with one copy of Cre recombinase from the Bl6 background (Fig. 1B). Animals from the SV background with two copies of Cre recombinase showed increased spleen weight [U=39, SV mutant with one copy of Cre recombinase (n_Het_)=13, SV mutant with two copies of Cre recombinase (n_Homo_)=15, *P*<0.01], increased WBC count (U=10, n_Het_=11, n_Homo_=10, *P*<0.05) and decreased hemoglobin (U=8, n_Het_=11, n_Homo_=10, *P*<0.01), compared with animals with one copy of Cre recombinase (Fig. 1B). Importantly, no control animals developed leukemia or skin disease. In this model, both alleles of *RBP-J* must be inactivated in order to result in disease development (mutant animals with only one floxed *RBP-J* allele never develop disease and are indistinguishable from control animals). Thus, animals with one copy of Cre recombinase achieve deletion of both copies of *RBP-J*, similar to animals with two copies of Cre recombinase, suggesting that the difference in phenotype is due to an earlier deletion. Together, these data demonstrate a strain-dependent phenotype and, in addition, suggest that more-efficient deletion of *RBP-J* within renin progenitors accelerates disease development and severity.

### Dermatitis/MPD phenotype in SV mice is due to cell-autonomous deletion of *RBP-J* in progenitor cells of the skin

**Fig. 2.**
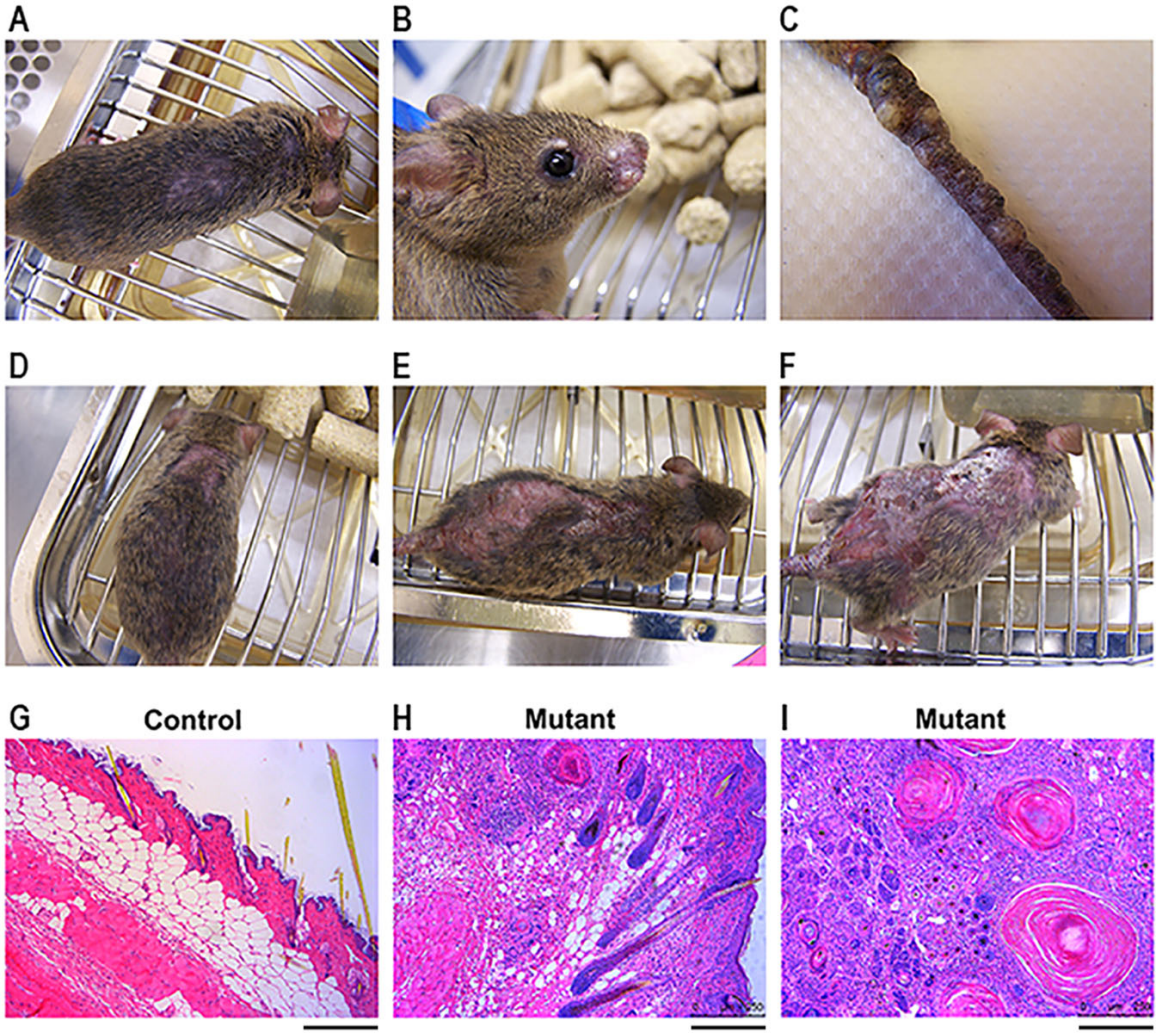
**Deletion of *RBP-J* within renin cells of SV mice leads to dermatitis.** (A-C) Mutant animals from the SV background develop hair loss over their back (A) and cystic nodules on their snouts (B) and tails (C). (D-F) With age, hair loss (D) progresses to ulceration and hyperkeratinization (E,F). Pictures are of the same mous­e at day of life 57 (D), day of life 107 (E) and day of life 155 (F). (G-I) Hematoxylin and Eosin staining demonstrates thickened epidermis and dermis, as well as large cysts and inflammatory infiltrates, in mutant animals (H,I) compared with control animals (G). Scale bars: 200 µm.

Animals from the SV background developed a severe dermatitis following conditional deletion of *RBP-J* within renin-expressing cells. Whereas Bl6 animals developed mild skin lesions most commonly consisting of fur loss in small patches [as previously described by Belyea et al. (2014)], mice from a SV background developed severe skin lesions with ulcerations and nodules. Affected animals typically presented at 2-4 months of age with hair loss over their backs and/or cystic nodules on their snouts, vibrissa regions and tails (Fig. 2A-C). With age, the hair loss progressed to hyperkeratinization of the epidermis and ulcerations (Fig. 2D-F). Histologic analysis of skin lesions stained with Hematoxylin and Eosin demonstrated thickened epidermis and dermis, large cysts in the dermal layer and extensive inflammatory infiltrates (Fig. 2G-I).

It was not initially clear whether these skin lesions occurred due to infiltration of the skin by neoplastic hematopoietic cells possessing deletion of *RBP-J*, or rather due to cell-autonomous deletion of *RBP-J* within skin progenitor cells. To answer this question, we performed lineage tracing in mutant mice with deletion of *RBP-J* in renin cells. In order to track the lineage of cells having undergone Cre-mediated deletion of *RBP-J*, we bred an mTmG reporter mouse to our mutant and control animals. At baseline, mTmG animals express RFP in all nucleated cells, but, in the presence of Cre recombinase, cells undergo deletion of RFP and induction of GFP. Thus, in our mutant animals (*RBP-J^fl/fl^;Ren1^dCre/+^;mTmG*), cells that express Cre recombinase (renin-expressing cells) will both delete *RBP-J* and turn on GFP.

**Fig. 3.**
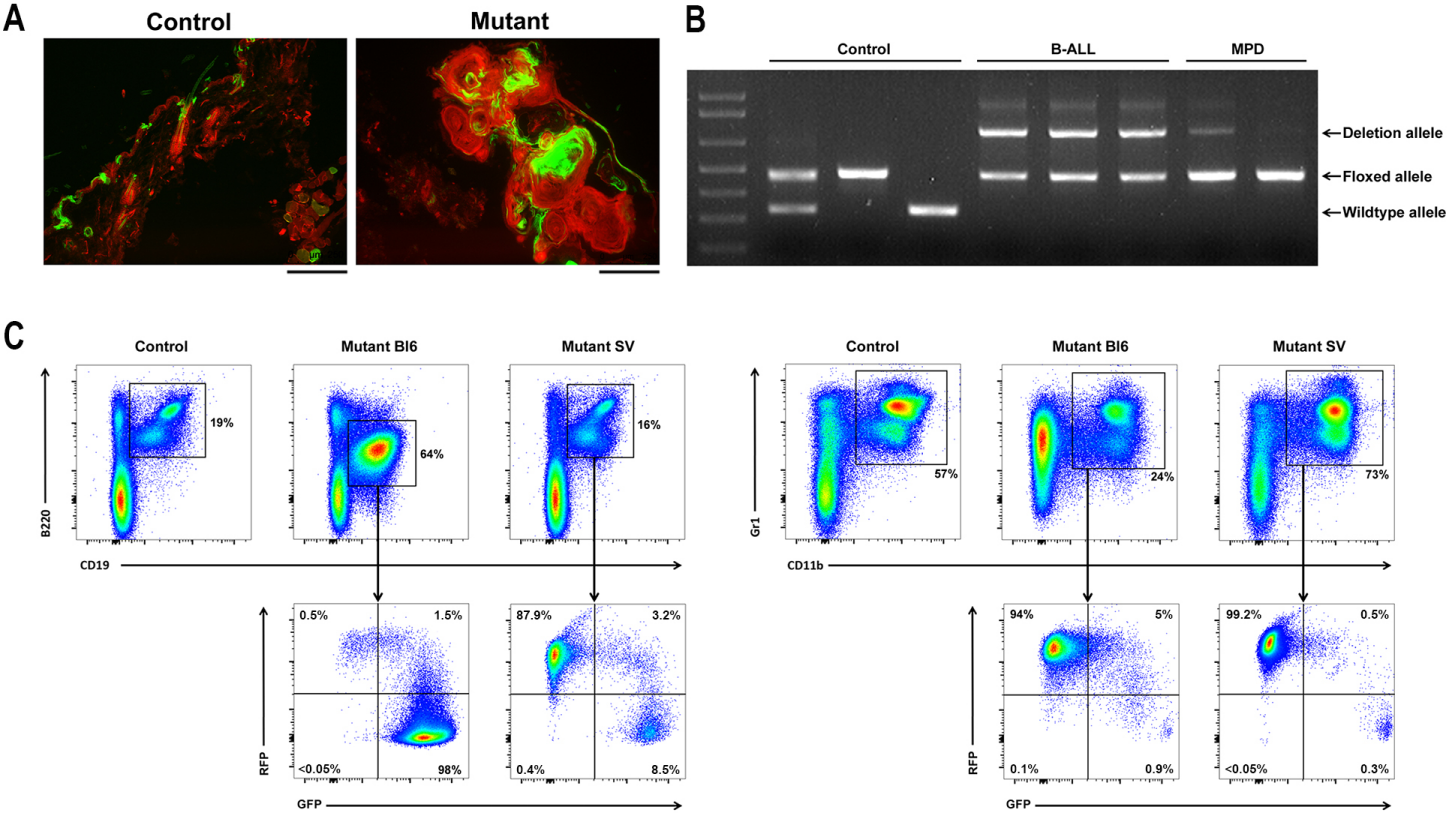
**Dermatitis in SV mice is due to deletion of *RBP-J* within progenitor cells of the skin and the MPD is a secondary, reactive process.** (A) Renin lineage (GFP^+^) cells are present in the skin of *RBP-J^fl/+^;Ren1^dCre/+^(SV);mTmG* control mice (left). The nodular and cystic skin lesions of *RBP-J^fl/fl^;Ren1^dCre/+^(SV);mTmG* mutant mice are partially GFP positive, signifying the presence of Cre recombinase and thus deletion of *RBP-J* within skin lesion cells (right). Scale bars: 200 μm. (B) PCR was performed on genomic DNA from bone marrow cells from control animals, animals with B-cell leukemia and animals with dermatitis/MPD, using primers designed to detect wild-type *RBP-J* alleles, floxed *RBP-J* alleles and *RBP-J* alleles that have undergone *Cre*-mediated deletion. The bone marrow cells from animals with MPD show predominantly floxed *RBP-J* alleles, but not deletion alleles, confirming that the myeloid cells have not undergone deletion of *RBP-J*. (C) Flow cytometry on bone marrow cells from mutant Bl6 animals shows a large population of CD19^+^B220^dim^ leukemia cells, which are largely GFP positive (left panel). Conversely, the bone marrow from mutant SV mice has an increased population of CD11b^+^Gr1^+^ myeloid cells, which are RFP positive (right panel), and thus have not undergone Cre-mediated recombination of *RBP-J*.

In mice that develop a dermatitis/MPD phenotype, we found that skin lesions were partially GFP positive, demonstrating the presence of Cre recombinase and thus deletion of *RBP-J* within skin cells (Fig. 3A). Conversely, the MPD appears to occur as a non-cell-autonomous process. Bone marrow cells from control and mutant animals were obtained and the status of the *RBP-J* gene was characterized by PCR. Using primers designed to detect wild-type, floxed and deleted alleles of *RBP-J*, PCR resulted in three different sized bands. We found that bone marrow cells from animals with dermatitis/MPD phenotype had predominantly floxed *RBP-J* alleles, but not deletion alleles, confirming the non-cell-autonomous nature of the myeloid proliferation (Fig. 3B). Further, we found that the increased population of monocytes and granulocytes in these animals did not express GFP by direct visualization (data not shown) and by flow cytometry, suggesting that Cre recombinase was not active and *RBP-J* was not deleted in these hematopoietic cells (Fig. 3C). These results are in contrast to Bl6 animals with deletion of *RBP-J* in renin cells, which develop B-cell leukemia with recombined (deleted) *RBP-J* alleles (as shown by PCR) and are GFP^+^ (as shown by flow cytometry) (Fig. 3B,C).

To test the hypothesis that the dermatitis was a result of cell-autonomous deletion of *RBP-J* within skin cells, we performed transplant studies. Mutant animals [*RBP-J^fl/fl^;Ren1^dCre/+^(SV)*] were transplanted with bone marrow cells from wild-type donor animals with an mT reporter construct. As a control, a second group of mutant animals was not transplanted. The animals were then monitored for worsening skin disease and other outward signs of poor health. Animals were studied when they became moribund or when their condition (dermatitis, weight loss, etc.) deteriorated. Engraftment of donor hematopoietic cells was confirmed as >80% in all transplanted animals by counting the percentage of peripheral blood WBCs that expressed mTd. We found no difference in the development of dermatitis or survival in mutant mice undergoing bone marrow transplant (Fig. S1A,B). We did, however, observe a trend towards decreased splenomegaly, hepatomegaly and leukocytosis in transplanted animals, suggesting that bone marrow transplant rescued the secondary myeloproliferation that accompanies the skin dermatitis (Fig. S1C).

Together, these data demonstrate that the dermatitis in mutant mice of the SV strain is caused by deletion of *RBP-J* within skin progenitor cells, and that the associated MPD is a reactive process, not due directly to the cell-autonomous deletion of *RBP-J* within hematopoietic cells.

### Bl6 mice have more renin lineage cells within hematopoietic tissues than SV mice

**Fig. 4.**
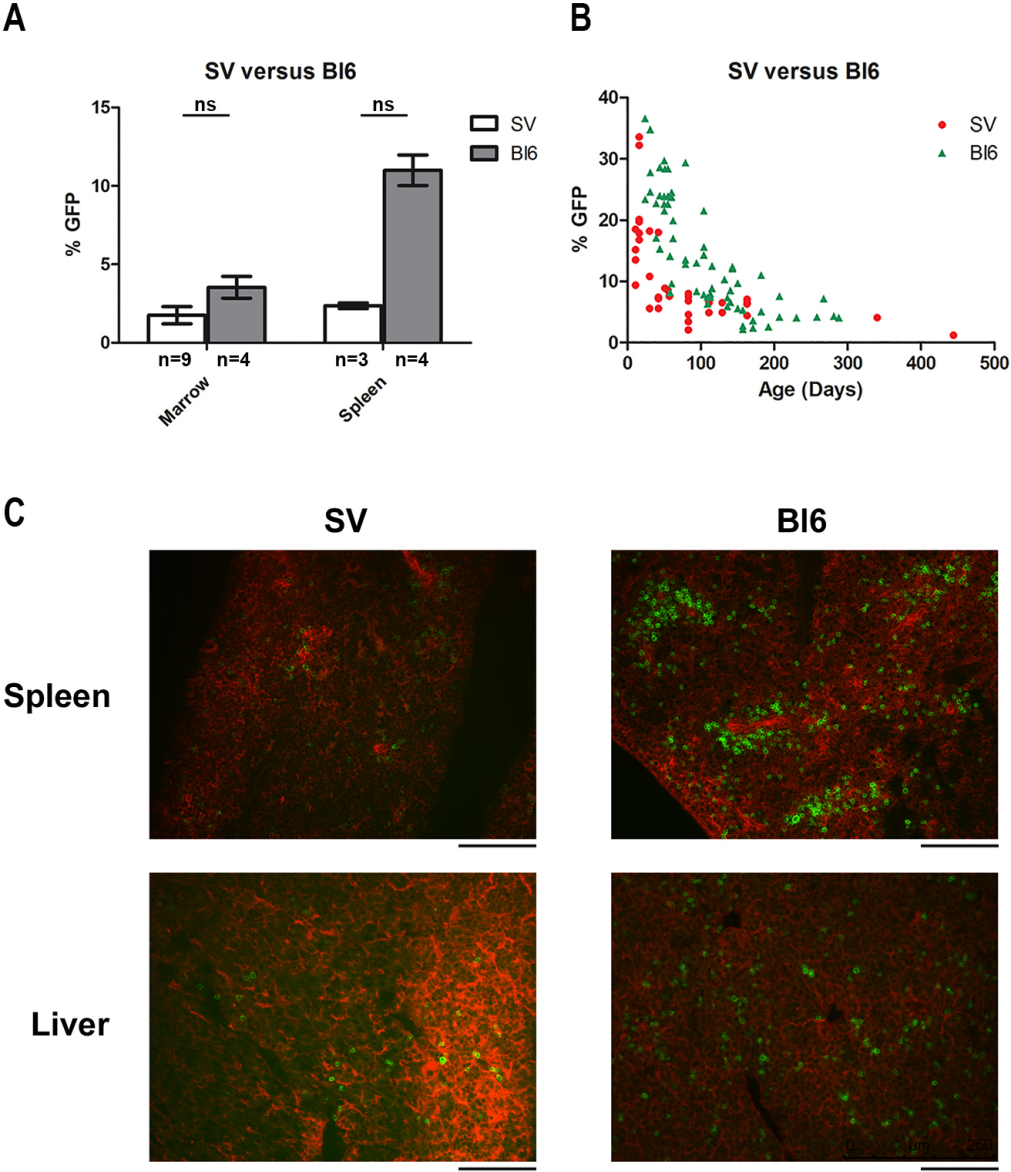
**Bl6 mice have more renin lineage cells within hematopoietic tissues compared with SV mice.** (A) Bl6 mice have a trend towards more renin lineage cells (marked by GFP) in the spleen compared with SV mice (*P*=0.057, Mann–Whitney *U*-test). There is a non-significant trend towards more renin lineage cells in the bone marrow as well (*P*=0.076, Mann–Whitney *U*-test). The sample size for each group is shown. (B) There are more renin lineage cells (GFP^+^) in the peripheral blood of Bl6 mice compared with SV mice throughout life. (C) Immunofluorescent images of renin lineage (GFP^+^) cells in the spleen and liver of newborn SV mice compared with newborn Bl6 mice. Scale bars: 100 µm.

To understand the difference in the incidence of B-cell leukemia between Bl6 and SV mice, we evaluated the number of renin lineage cells within hematopoietic tissues of wild-type mice from both strains. First, we performed lineage tracing using flow cytometry on our *Ren1^dCre^;mTmG* reporter mice. We found a trend towards higher numbers of renin lineage cells in the bone marrow (*P*=0.076) and spleen (*P*=0.057) of Bl6 mice compared with SV mice at 4 months of age (Fig. 4A). Next, we determined the percentage of renin lineage cells in the peripheral blood of SV and Bl6 mice from early life (<1 month of age) to adulthood (up to 10 months of age). We found that at younger ages (0-3 months), there were more renin lineage cells in the blood of Bl6 mice compared with SV mice (U=120.5, n_Bl6_=28, n_SV_=26, *P*<0.001) (Fig. 4B; Fig. S2). Finally, we performed immunofluorescence imaging of the liver and spleen of newborn SV and Bl6 mice and found that Bl6 mice had higher numbers of renin lineage cells (Fig. 4C). Together, these data demonstrate that Bl6 mice have increased numbers of renin lineage cells within the hematopoietic system compared with SV mice.

### Conditional deletion of *RBP-J* in Mb1- and CD19-expressing cells does not cause B-cell leukemia

To test the hypothesis that renin-expressing cells, as opposed to other cells from the B-lymphoid lineage, constitute a special subgroup of hematopoietic progenitor cells that exhibit enhanced malignant potential, we designed the following experiments. We generated two independent groups of mice with conditional *RBP-J* deletion using different B-cell stage-specific *Cre* transgenes. Specifically, we deleted *RBP-J* in Pro-B cells (*Mb1-Cre*) (Hobeika et al., 2006) and Pre-B cells (*CD19-Cre*) (Rickert et al., 1997). To generate conditional knockout mice, we first crossed *RBP-J^fl/fl^* mice with EIIa-Cre mice, which express Cre recombinase in the early embryo, resulting in germline deletion of the loxP-flanked *RBP-J* allele (*RBP-J^del/+^*) (Lakso et al., 1996). These mice were then crossed with *RBP-J^fl/fl^* mice and mice with Cre recombinase (Ren1^d^, Mb1 or CD19), ultimately generating mice with one deleted *RBP-J* allele, one floxed *RBP-J* allele and one copy of Cre recombinase. To determine the percentage of cells that express Cre recombinase, and to track the lineage of cells that have undergone deletion of *RBP-J*, we bred our control and mutant mice with *mTmG* reporter mice, which constitutively express RFP in all cells that do not express Cre recombinase and GFP only in cells that express Cre recombinase (Muzumdar et al., 2007).

**Fig. 5.**
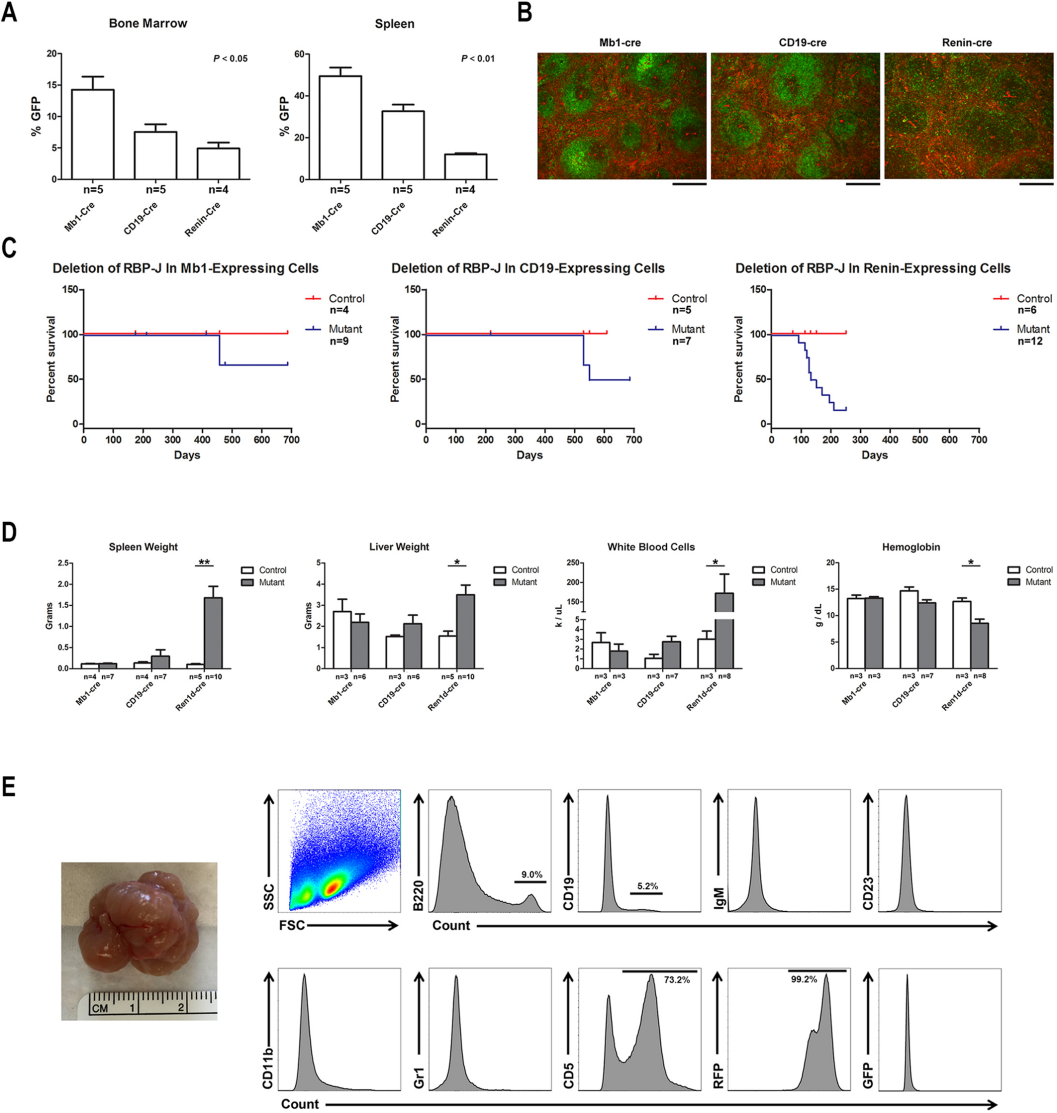
**Renin-expressing cells are a susceptible population for malignant transformation.** (A) Comparison of the expression pattern of different B-cell Cre recombinase transgenes – Mb1-Cre, CD19-Cre and renin-Cre – within the bone marrow and spleen by flow cytometry. Within the bone marrow and spleen, *Mb1^Cre^;mTmG* mice have the highest number of B cells that express GFP, followed by *CD19^Cre^;mTmG* and *Ren1^dCre^;mTmG*. *P<0.05 and **P<0.01, Kruskal–Wallis test. The sample size for each group is shown. (B) Cre-mediated GFP expression in the spleens of *Mb1^Cre^;mTmG, CD19^Cre^;mTmG* and *Ren1^dCre^;mTmG* mice. Scale bars: 200 µm. (C) Conditional deletion of RBP-J within renin-expressing cells leads to the development of B-cell leukemia and early death. However, conditional deletion of *RBP-J* within Mb1-expressing or CD19-expressing cells does not lead to B-cell leukemia. At advanced ages, 20% of *RBP-J^del/fl^;Mb1^Cre/+^* mice and 50% of *RBP-J^del/fl^;CD19^Cre/+^* mice developed abdominal tumors, leading to abdominal distension and death. The sample size for each group is shown. (D) Conditional deletion of *RBP-J* within renin-expressing cells, but not Mb1-expressing or CD19-expressing cells, resulted in elevated spleen weight, liver weight and white blood cell count and decreased hemoglobin compared with littermate control mice. *P<0.05 and **P<0.01, Mann–Whitney *U*-test. The sample size for each group is shown. (E) Representative tumor from *RBP-J^del/fl^;CD19^Cre/+^* mice (leftmost). By flow cytometry analysis, abdominal tumors were positive for CD5 but negative for B-cell and myeloid markers. Further, the tumors were RFP^+^ and thus not due directly to deletion of *RBP-J*. FSC, forward scatter; SSC, side scatter.

First, we determined the percentage of cells within the bone marrow and spleen that derive from the different precursors, as measured by expression of GFP (Fig. 5A). In the bone marrow, *Mb1^Cre^;mTmG* mice have the highest percentage of B cells that express GFP (*n*=5), followed by *CD19^Cre^;mTmG* (*n*=5) and *Ren1^dCre^;mTmG* (*n*=4) (Kruskal–Wallis statistic (H)=8.69, *P*<0.05). There was a similar pattern in the spleen (H=10.52, *P*<0.01). These findings are consistent with prior reports demonstrating that Mb1-Cre is more highly expressed than CD19-Cre in early B cells (Hobeika et al., 2006; Rickert et al., 1997). As expected, there was a higher percentage of GFP^+^ cells in the spleen than in the bone marrow, consistent with known patterns of B-cell development (Fig. S3). Second, we let mice with conditional deletion of *RBP-J* age and monitored them for signs and symptoms of leukemia/lymphoma development. Specifically, we performed complete blood counts, peripheral blood smear analysis, flow cytometry on bone marrow samples, and necropsy and histological analysis on mutant and control animals. Whereas animals with conditional deletion of *RBP-J* within renin-expressing cells developed B-cell leukemia at a high frequency (as previously reported and discussed above), no animals with deletion of *RBP-J* in Mb1- or CD19-expressing cells developed B-cell leukemia (Fig. 5C,D). Animals with deletion of *RBP-J* in either Mb1- or CD19-expressing cells demonstrated a mild decrease in the B220^+^CD19^+^ B-cell population within the bone marrow, which was more evident at older ages (data not shown). Of note, a significant percentage of animals (20% in Mb1 mice and 50% in CD19 mice) developed abdominal tumors at very old age (>1 year) (Fig. 5E). These tumors exhibited forward scatter and size scatter patterns consistent with lymphocyte populations and were negative for B-cell and myeloid markers, including B220, CD19, IgM, CD23, CD11b and Gr1. Tumors were, however, positive for CD5, suggesting a T-cell origin for these tumors. Finally, all of these tumors were RFP^+^ and thus not due directly to deletion of *RBP-J* (Fig. 5E). This result was confirmed by PCR studies showing the abdominal tumors had not undergone deletion of *RBP-J* (data not shown).

## DISCUSSION

In this work, we used conditional gene deletion models based on the Cre-*loxP* system, lineage tracing, flow cytometry and histologic analysis to further characterize a genetic mouse model of B-cell leukemia. We found remarkable differences in leukemia incidence, severity and phenotype based on the strain of mouse, cell of origin and deletion efficiency.

First, we found two unique disease phenotypes that occurred in a strain-dependent manner. Conditional deletion of *RBP-J* in mice from a Bl6 background resulted in a high incidence of B-cell leukemia, characterized by massive organomegaly, leukocytosis and a B220^dim^CD19^+^ bone marrow immunophenotype. Conversely, conditional deletion of *RBP-J* in SV mice led to a primary dermatitis consisting of severe, ulcerating lesions as well as mild organomegaly, leukocytosis and a myelomonocytic bone marrow phenotype. Leukemia cells from Bl6 mice contain cell-autonomous deletion of *RBP-J*, whereas myeloid cells from SV mice have intact *RBP-J* gene based on PCR and lineage-tracing studies. The reason for this difference in phenotype is not completely clear. Bl6 animals have increased numbers of renin lineage cells throughout the hematopoietic system, including the bone marrow, spleen and peripheral blood. Thus, it may be that Bl6 animals have more renin-expressing hematopoietic cells undergoing deletion of *RBP-J* at an earlier age, and leukemia development in this model occurs in a time-dependent manner. This possible mechanism is supported by our studies showing that more efficient deletion of *RBP-J* in renin-expressing cells results in a higher incidence of B-cell leukemia: in both SV and Bl6 strains, animals with two copies of Cre recombinase had a higher incidence of B-cell leukemia as well as a shorter latency to leukemia development. Thus, the incidence and timing of leukemia development in this model are sensitive to the efficiency of *RBP-J* deletion within renin-expressing cells. In addition, it remains possible that there is a modifier gene(s) in C57BL/6 or 129/SV mice that accelerates or suppresses the development of leukemia, respectively. Indeed, a phenotypic difference between C57BL/6 and 129/SV mice due to genetic modifiers has previously been described, leading to strain-specific embryo susceptibilities to *p300* (also known as *Ep300*) and *cbp* (also known as *Crebbp*) gene deletions (Yao et al., 1998). Finally, we cannot exclude the possibility that decreased levels of circulating renin plays a contributory role in disease development, as the Ren1^dCre^ Cre recombinase is a knock-in transgene, which replaces the intact renin gene. Thus, animals with two copies of Cre recombinase will have both renin genes deleted. Further, RBP-J is critical for renin expression, as deletion of *RBP-J* in renin cells leads to decreased renin levels (Castellanos Rivera et al., 2011). Together, these two factors lead to decreased renin expression, which may contribute to the leukemia model in a strain-specific manner.

In addition to lymphopoiesis, the Notch signaling pathway plays a critical role in cell-fate decisions within skin progenitor cells. Previous studies have shown that deletion of Notch signaling within skin progenitor cells leads to skin barrier defects, including hair loss, epidermal thickening, hyperkeratinization and epidermal cyst formation (Yamamoto et al., 2003; Rangarajan et al., 2001). In our work, we found the presence of renin-lineage cells within the skin of both Bl6 and SV mice. Based on our studies, we believe that the skin lesions found in mutant animals are due to Cre-mediated deletion of *RBP-J* in renin-expressing precursor cells of the skin. These defects in skin differentiation may lead to production of cytokines, triggering an inflammatory response and ultimately resulting in a secondary, reactive monocyte and granulocyte expansion in the bone marrow. A similar phenotype has been seen in animals with conditional deletion of *RBP-J* within skin cells using Cre recombinase under the control of the *Krt5* promoter (Dumortier et al., 2010). In their work, Dumortier et al. showed that deletion of the Notch signaling pathway within keratin-expressing skin cells leads to a severe atopic dermatitis-like disease, which is accompanied by a lethal non-cell-autonomous MPD. Although their animals developed more severe dermatitis with the entire skin undergoing deletion of *RBP-J*, the sequence of dermatitis followed by MPD is similar to our model. That skin progenitors express renin was an unexpected finding, although recent work by our laboratory and others have demonstrated renin expression by multiple extra-renal progenitor cells (Sequeira López et al., 2004; Glenn et al., 2014; Gomez and Sequeira-Lopez, 2016). Why skin progenitor cells express renin is not currently known; however, we suspect that renin might be involved in primitive defense mechanisms of the skin and/or that a local skin renin-angiotensin system might be involved in skin regeneration and wound healing.

The Notch signaling pathway plays critical roles during normal lymphocyte development, including progenitor cell-fate decisions. Prior studies have demonstrated that overexpression of the Notch signaling pathway in hematopoietic progenitors stimulates T-cell development and blocks B-cell differentiation (Pui et al., 1999). Conversely, inhibition of Notch signaling in lymphocyte progenitors blocks T-cell development (Radtke et al., 1999) and enforces ectopic B-cell development (Wilson et al., 2001). Thus, the normal role of Notch/RBP-J signaling during lymphocyte development is to promote T-cell commitment over B-cell fate from common lymphocyte progenitors, and disruption of normal Notch signaling in these cells can lead to malignant transformation. Here, we demonstrate that deletion of Notch signaling within pro- and pre-B cells (using Mb1- and CD19-driven Cre recombinase, respectively) leads to a high incidence of T-cell lymphoma. The T-cell lymphomas developed late in life and occurred in a non-cell-autonomous manner (the *RBP-J* gene was intact). The mechanisms of T-cell lymphoma development in these mice remain unclear; however, it is possible that deletion of Notch signaling in B cells (GFP^+^ cells) leads to generalized disruption of Notch signaling throughout the bone marrow compartment with subsequent upregulation of Notch signaling within T lymphocytes, ultimately leading to pathologic cell transformation of T cells (RFP^+^).

In summary, we show that conditional deletion of *RBP-J* in renin-expressing cells leads to B-cell leukemia in Bl6 mice and dermatitis/MPD in SV mice. The final disease phenotype depends on mouse strain, *RBP-J* deletion efficiency and the cell/tissue of origin. These findings raise several important questions, including the function of renin within skin and B-cell progenitors and the increased susceptibility of renin progenitors to both malignant and cystic/proliferative transformation. Further, this work reinforces the idea that disease models need to be carefully examined for confounding factors that might not be universally valid among all model systems. In humans, the development of B-cell leukemia requires genetic and/or epigenetic changes that occur within a vulnerable cell of origin. However, there are likely many other factors that contribute to the final disease phenotype including a conducive bone marrow environment, selective stresses such as infection, and some still unknown ones. A furthered understanding of these contributing factors will advance our knowledge of leukemia genesis and ultimately promote the development of improved strategies for the detection and treatment of this malignant disease.

## MATERIALS AND METHODS

### Generation of study mice

To inactivate RBP-J in renin lineage cells, we crossed *Ren1^dCre/+^* mice, which express Cre recombinase in renin cells, to *RBP-J* floxed mice (Han et al., 2002; Sequeira López et al., 2004). Mice carrying both *RBP-J^fl/fl^* and *Ren1^dCre/+^* undergo Cre-mediated recombination specifically in cells that express renin, leading to deletion of *RBP-J*. To evaluate *RBP-J* deletion efficiency, we generated animals with one (*RBP-J^fl/fl^;Ren1^dCre/+^*) or two (*RBP-J^fl/fl^;Ren1^dCre/Cre^*) copies of Cre recombinase. Animals with one wild-type *RBP-J* allele (*RBP-J^fl/+^;Ren1^dCre/+^*) or lacking Cre recombinase (*RBP-J^fl/fl^;Ren1^d+/+^*) mice were used as controls. To inactivate RBP-J in pro-B cells and pre-B cells, *RBP-J^fl/fl^* mice were crossed with *Mb1^Cre/+^* or *CD19^Cre/+^* mice, respectively. We crossed *RBP-J^fl/fl^* mice with EIIa-Cre mice, which express Cre in the early embryo, resulting in germline deletion of the loxP-flanked *RBP-J* allele (*RBP-J^del/+^*) (Lakso et al., 1996). These mice were then crossed with *RBP-J^fl/fl^* mice and either *Ren1^dCre/+^*, *Mb1^Cre/+^* or *CD19^Cre/+^* mice, ultimately generating mice with one deleted *RBP-J* allele, one floxed *RBP-J* allele and one copy of Cre recombinase (Table S1). Mutant mice were monitored until they developed signs/symptoms of disease. When mice became moribund, they were anesthetized with tribromoethanol (300 mg/kg), and organs were removed, preserved for RNA extraction, fixed for immunohistochemistry or immunofluorescence, or processed for flow cytometry. Blood was collected by cardiac puncture. *Ren1^dCre^* animals were generated previously in our laboratory (Sequeira López et al., 2004), *RBP-J^fl/fl^* mice were obtained from T. Honjo (Kyoto University, Kyoto, Japan), and *Mb1^Cre^* and *CD19^Cre^* mice were obtained from T. Bender (University of Virginia, Charlottesville, VA, USA). All procedures were performed in accordance with the National Institutes of Health Guide for the Care and Use of Laboratory Animals and were approved by the Animal Care and Use Committee of the University of Virginia.

### Genotyping

Tail biopsies were sent to Transnetyx for genotyping (Transnetyx Genotyping Services, Cordova, TN, USA).

### Lineage tracing

Lineage tracing was performed to determine the pattern of renin expression and track Cre recombinase activity. *Ren1^dCre/+^* mice were bred to the lineage reporter line *mT/mG*, in which non-recombined cells express mTd and Cre-mediated recombined cells express GFP (Muzumdar et al., 2007). Thus, GFP is expressed in renin-expressing cells and all descendants. A similar strategy was used for Mb1-Cre and CD19-Cre mice.

### Flow cytometry

To characterize the immunophenotype of hematopoietic cells from mutant and control mice, flow cytometry analysis was performed. Single-cell suspensions were obtained from the bone marrow, spleen and abdominal tumors. Cells were obtained as previously described (Belyea et al., 2014). Briefly, the femur and tibia of mice were dissected, flushed with PBS+5% fetal bovine serum (FBS), and cells were passed through a 70-µm cell strainer. Spleen and tumor samples were obtained by passing the tissue through a 70-µm cell strainer, using a syringe plunger to gently disrupt the tissues. Cells were rinsed through the strainer with PBS/FBS. Then cells were treated with red blood cell lysis buffer, resuspended in PBS/FBS, counted using the Cellometer mini (Nexcelom Bioscience, Lawrence, MA, USA) and distributed into microcentrifuge tubes for antibody labeling against surface markers (Table S2). Antibodies were added at predetermined optimum concentrations and incubated at room temperature for 20 min. Immunophenotyping was performed in the University of Virginia Flow Cytometry Core laboratories using a Fortessa cytometer and data were analyzed with the FlowJo program (FlowJo, Ashland, OR, USA).

### RNA extraction and polymerase chain reaction analysis

Total RNA was isolated from the bone marrow and spleen samples using Trizol extraction (Life Technologies, Grand Island, NY, USA) according to the manufacturer's instructions. Complementary DNA (cDNA) was prepared from 2 µg RNA using Moloney murine leukemia virus reverse transcriptase (Life Technologies) and an oligo(dT) primer according to the manufacturer's instructions. PCR was performed on 2 µl cDNA using Taq DNA polymerase (Promega, Madison, WI, USA) in an Eppendorf thermocycler. We used a combination of three primers to detect three different *RBP-J* alleles (wild-type, floxed and recombined): 5′-TTTGCTTGAGGCTTGATG-3′, 5′-GGTTTGTTGTTTGGGTTG-3′ and 5′-CATTTTGTACTCACAGAGATG-3′, respectively.

### Histologic analysis

Tissues were harvested and fixed overnight in either Bouin's solution or formalin and embedded in paraffin. Tissue sections (5 µm) were deparaffinized in xylenes and graded alcohols and stained with Hematoxylin and Eosin to examine tissue morphology. Immunofluorescence was performed on frozen sections.

### Transplant studies

Bone marrow from reporter mice with the mTd reporter were transplanted into *RBP-J^fl/fl^;Ren1^dCre/+^* mutant mice from the SV strain after being treated with radiation (13 Gy divided into two fractions). Mutant mice had early signs of dermatitis prior to transplant. Successful transplant was confirmed by the presence of *mTd* in the transplant recipient animals (>80% of nucleated blood cells positive for *mTd*). Transplanted animals were monitored for signs/symptoms of worsening dermatitis.

### Statistical analysis

Specific tests are noted in the text and figure legends. Statistical significance between groups was evaluated using the Mann–Whitney *U*-test, Kruskal–Wallis test with Dunn's multiple comparison post-test or the Fisher's exact test. Differences were considered statistically significant at *P<0.05, **P<0.01 and ***P<0.001 levels. Bar graphs are expressed as mean±s.e.m.

## Supplementary Material

Supplementary information
