# Supplementary material for: Leukemia development initiated by deletion of RBP-J: mouse strain, deletion efficiency and cell of origin
Source: Dis Model Mech. 2018 Dec 18;11(12):dmm036731. doi: 10.1242/dmm.036731 (PMC6307899; doi:10.1242/dmm.036731)
Supplement: Supplementary information [file dmm-11-036731-s1.pdf]

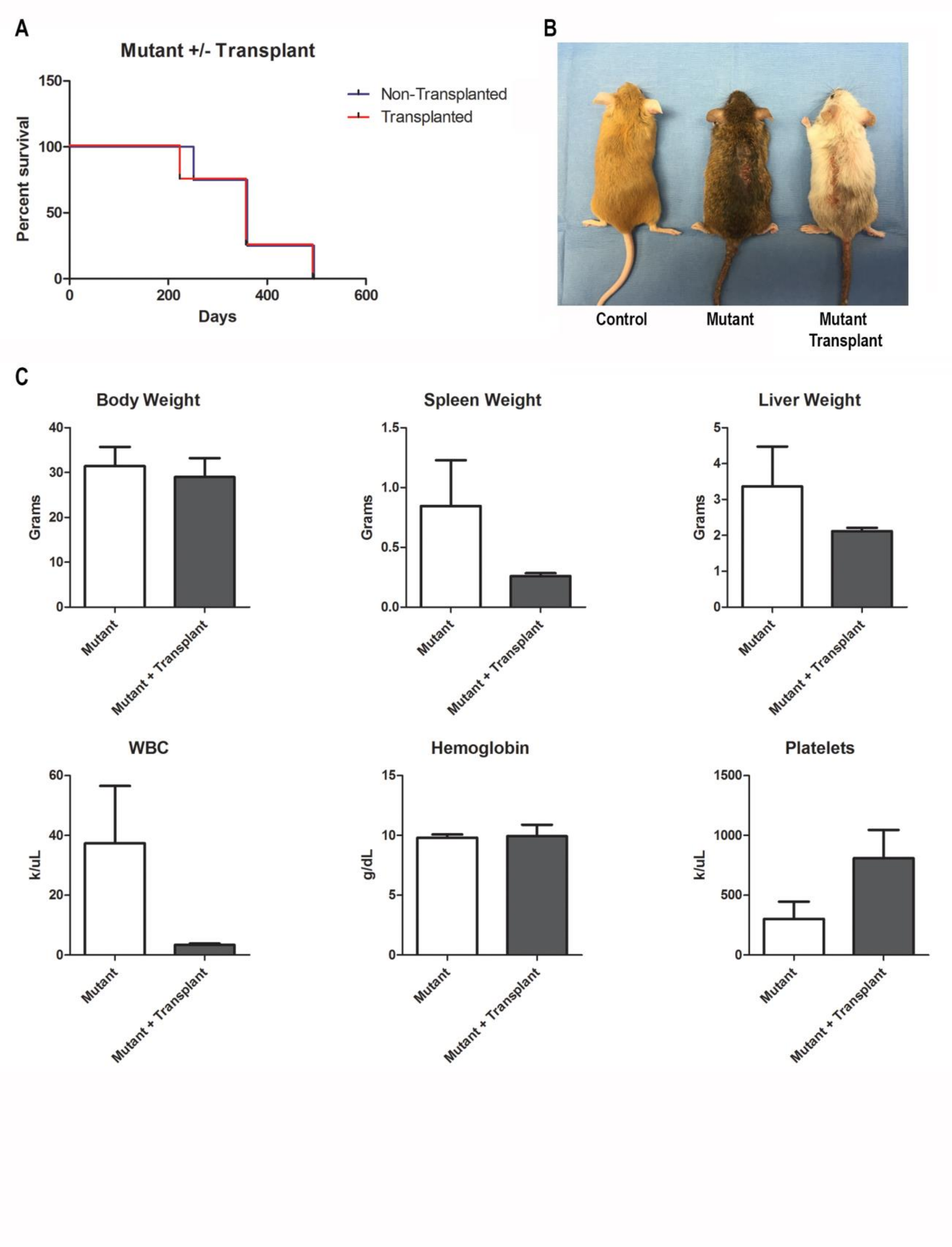

Disease Models & Mechanisms • Supplementary information

Figure S1. The dermatitis phenotype in mutant animals from the SV background is the result of cell-autonomous deletion of RBP-J within skin cells and cannot be rescued by bone marrow transplant.

A: Four mutant animals from the SV background (*RBP-J*$^{fl/fl}$;*Ren1*$^{dcre/+}$*(SV)*) were transplanted with bone marrow cells from wildtype donor animals. Age- and disease-matched mutant animals were selected as controls and were not transplanted. Transplant did not rescue the dermatitis, and there was no difference in survival between these groups.

B: Representative pictures following transplant. The skin phenotype of mutant mice did not improve following bone marrow transplant, suggesting that the dermatitis is not secondary to the myeloproliferative process in the bone marrow.

C: Bone marrow transplant in mutant mice may lessen the myeloproliferative process in the bone marrow.

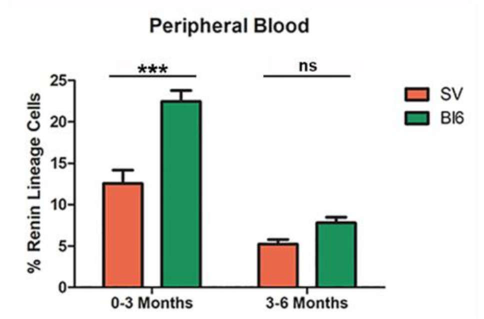

Figure S2. Mice from a Bl6 background have more renin-lineage cells in their blood during the first 3 months of life compared to mice from a SV background.

The percentage of renin-lineage (GFP+) cells in the blood was determined at different post-natal ages (same data as shown in Figure 4B). In order to perform statistical analysis, we grouped the data into two “bins” representing ages 0-3 months and 3-6 months. There are more renin-lineage cells in the peripheral blood of Bl6 mice compared to SV mice at ages <3 months of age (7.8 ± 0.69 % n=37 versus 5.24 ± 0.56 % n=10, $P < 0.001$).

Disease Models & Mechanisms • Supplementary information

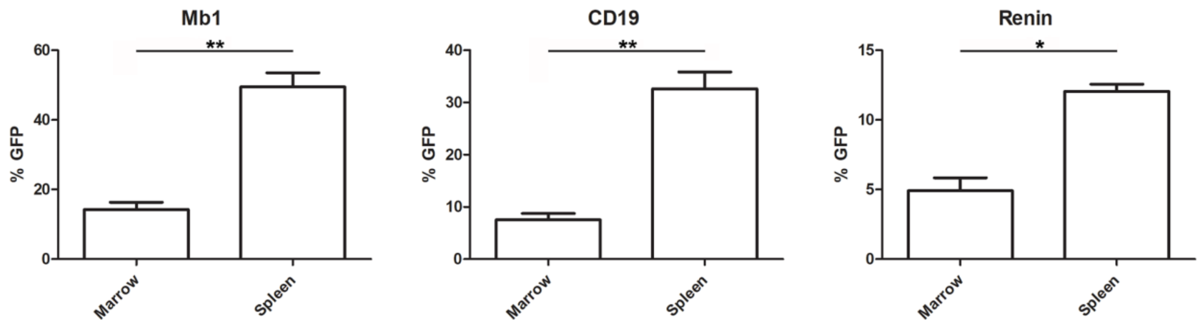

Figure S3. There are more GFP+ cells in the spleen compared to bone marrow of transgenic mice.

The percentage of GFP+ cells within the bone marrow and spleen from different B cell stage-specific cre transgenic mice was determined including Pro B-cells (*Mb1-cre*), Pre B-cells (*CD19-cre*), and Renin-expressing B-cells (*Renin-cre*). In all three groups, there were more GFP+ cells in the spleen compared to the bone marrow. Mann-Whitney U test, $*P < 0.05$ and $**P < 0.01$.

Table S1. Conditional deletion of RBP-J using different cre recombinase transgenes.

Genotype	Copies of Cre	Site of cre expression
$RBP\text{-}J^{fl/fl};Ren1^{dcre/+}$	1	Renin-expressing cells
$RBP\text{-}J^{fl/fl};Ren1^{dcre/cre}$	2	Renin-expressing cells
$RBP\text{-}J^{del/fl};Ren1^{dcre/+}$	1	Renin-expressing cells
$RBP\text{-}J^{del/fl};Mb1^{cre/+}$	1	Pro B cells
$RBP\text{-}J^{del/fl};CD19^{cre/+}$	1	Pre B cells

Table S2. Antibodies used in flow cytometry analysis.

Antibody		Fluorochrome	Concentration	Catalogue # (Biolegends)
B220	Pan B cell marker expressed from pro-B cells through mature B cells	APC/Cy7	1 µg per 10^6 cells	103223
CD5	Expressed on T cells and a subset of B cells called B-1 cells	Brilliant Violet 421	0.25 µg per 10^6 cells	100617
CD11b	Expressed on granulocytes, monocytes, and macrophages	PerCP/Cy5.5	0.25 µg per 10^6 cells	101227
CD19	Pan B cell marker expressed from pro-B cells through mature B cells	Alexa Fluor 647	0.25 µg per 10^6 cells	115525
CD23	Expressed on mature B cells including follicular B cells	PE/Cy7	0.05 µg per 10^6 cells	101613
Gr1	Expressed on maturing granulocytes	PE/Cy7	0.05 µg per 10^6 cells	108415
IgM	Expressed on immature and mature B cells	Brilliant Violet 421	5 µl per 10^6 cells	406517

Disease Models & Mechanisms • Supplementary information
